# Trapped in the NETs: Multiple Roles of Platelets in the Vascular Complications Associated with Neutrophil Extracellular Traps

**DOI:** 10.3390/cells14050335

**Published:** 2025-02-25

**Authors:** Christopher Sennett, Giordano Pula

**Affiliations:** Biomedical Institute for Multimorbidity (BIM), Hull York Medical School (HYMS), University of Hull, Hull HU6 7RX, UK

**Keywords:** neutrophil extracellular trap, NET, NETosis, platelet

## Abstract

Neutrophil extracellular traps (NETs) have received significant attention in recent years for their role in both the immune response and the vascular damage associated with inflammation. Platelets have been described as critical components of NETs since the initial description of this physio-pathological response of neutrophils. Platelets have been shown to play a dual role as responders and also as stimulators of NETs. The direct interaction with DNA leads to the entrapment of platelets into NETs, a phenomenon that significantly contributes to the thrombotic complications of inflammation and neutrophil activation, while the direct and paracrine stimulation of neutrophils by platelets has been shown to initiate the process of NET formation. In this review, we provide a comprehensive description of our current understanding of the molecular mechanisms underlying the entrapping of platelets into NETs and, in parallel, the platelet-driven cellular responses promoting NET formation. We then illustrate established examples of the contribution of NETs to vascular pathologies, describe the important questions that remain to be answered regarding the contribution of platelets to NET formation and NET-dependent cardiovascular complication, and highlight the fundamental steps taken towards the application of our understanding of platelets’ contribution to NETs for the development of novel cardiovascular therapies.

## 1. Neutrophil Extracellular Traps (NETs): Essential Components of the Innate Immune Response or Pathological Alterations of the Vascular System?

**Figure 1:**
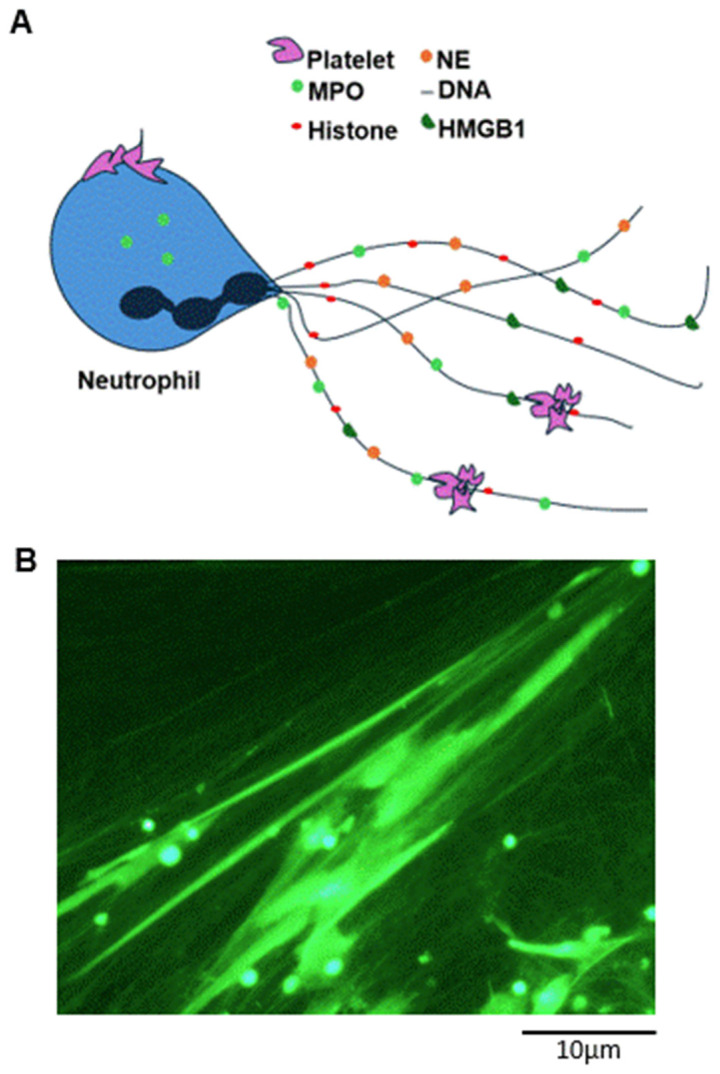
NET structure and composition. (**A**) A visual representation of NETs, including the fibrillary nature associated with the DNA content and well-characterised protein components, such as myeloperoxidase (MPO), histones, neutrophil elastase (NE), and high mobility group box 1 (HMGB1). (**B**) Fluorescence image of NETs, obtained by neutrophil isolation from whole blood, culture, and stimulation with PMA. DNA staining was obtained with Sytox Green.

Neutrophils are polymorphonuclear leukocytes (PMNs), the most abundant innate immune phagocytes comprising 50–70% of all white blood cells. Neutrophils were first discovered in 1883 by Elie Metchnikoff, and were observed to destroy pathogens through phagocytosis through the generation of reactive oxygen species (ROS) [1]. NETs are fibrous webs formed by extracellular DNA filaments associated with histones (H1, H2A, H2B, H3, and H4), myeloperoxidase (MPO), and secreted proteolytic enzymes, enzymatic Cathepsin G, Proteinase 3, calprotectin, cathelicidins, and defensins [2,3,4,5] (Figure 1). NETs trap and degrade pathogens thanks to the bactericidal activity of the enzymes they contain [3,6,7]. Although NETs are the most physiopathologically relevant and investigated, other cell types have also shown the ability to form extracellular traps; the different extracellular traps are described by their cellular origins: macrophage/monocyte extracellular traps are termed METs, mast cell extracellular traps are termed MCETs, and basophil extracellular traps are termed BETs [8].

The generation of NETs or NETosis was first reported in 1996 by Takei et al. [9], using phorbol myristate acetate (PMA) to induce neutrophil death. Further studies have demonstrated that, in addition to PMA, interleukin(IL)-1, -8, and -6, lipopolysaccharide (LPS), and IFN-r can also induce NET formation [10,11]. More recent studies have highlighted the importance of the combination of different paracrine factors and a prominent role for tumour necrosis factor-α (TNF-α) and lymphotoxin-α [12]. In addition, the stimulation with peptidyl–arginine deiminase 4 (PAD4) has been shown to citrullinate histones, favouring chromatin decondensation and leading to NET formation [13,14].

Platelets have been shown to interact with neutrophils and induce the formation of NETs; the surface markers P-selectin and GPIbα play a critical role in the interaction with neutrophils and the induction of NETs [15]. Additionally, paracrine factors released by platelets have been described for their role in the promotion of NETs; platelet factor 4 (PF4), CXCL4, regulated on activation normal T-cell expressed and secreted (RANTES), and high mobility group box 1 (HMGB1) have been shown to induce NETs in different experimental models [16,17,18,19]. Finally, platelet-derived exosomes have also been shown to promote the formation of NETs in response to sepsis, resulting in additional organ damage [20].

Early studies suggest that NETosis pathways culminate in cell death; however, recent studies suggest that there are two forms of NETosis: classical (suicidal NETosis), which leads to cell death, and vital NETosis, where the cell retains viability and many of its functions [21,22,23]. NETosis is distinct from apoptosis or necrosis and is characterised by distinctive morphological features, including the decondensation of chromatin and the disorganisation of cellular compartments [24,25]. PMA-induced NET formation is dependent on ROS generated by NADPH oxidases, which are activated by PKC [24]. However, other kinases such as c-Raf, MEK, AKT, and ERK have been shown to contribute to the activation of NADPH oxidases; these signalling cascades also stimulate the expression of Mcl-1, the main anti-apoptotic regulator in neutrophils [13]. Contrary to previous observations, studies have shown that mitochondria are key to NET formation [26]. It has been shown that mitochondrial ROS are involved in the induction of NETosis in response to multiple stimuli [26,27,28].

The formation and composition of NETs have been shown to vary in response to the mechanism/pathway triggered during formation. PMA stimulation of NETs induces classical NETosis through NADPH oxidase 2 (NOX2), whereas the formation of NETs through stimulation with peptidyl–arginine deiminase 4 (PAD4) citrullinates histones, favouring chromatin decondensation [13,14].

The mechanisms by which NETosis is stimulated in vivo, and how this may affect the phenotype generated, is poorly characterised. However, studies have shown that NET formation can be influenced by the presence of type I interferons, which display a proinflammatory phenotype in vivo [29]. Neutrophils are not just an important member of the innate immune system; a growing body of literature shows their involvement in the regulation of adaptive immunity through their interaction with various immune cells [30,31]. This is compounded by recent studies which have identified large phenotypic heterogeneity and differential neutrophil sub-types that change in stoichiometry among different diseases such as sepsis and cancer [32,33,34].

Although NETs are primarily associated with immunity, recent studies have elucidated the role of NETs in multiple pathologies, such as autoimmune disease [4,35], thrombotic occlusions and atherosclerosis [36,37], aseptic inflammation, coronavirus-disease-19 (COVID-19)-related thrombosis [38], and immune suppression in cancer [39]. NETs have been proposed to contribute to the development of thrombosis by forming a structure that induces platelet adhesion, activation, and aggregation [40]. Thrombi generated by NETs have a high red blood cell (RBC) content analogous to venous thrombi. In the first instance, this has been linked to the formation of DVT, an hypothesis supported by elevated plasma DNA levels [41]. Subsequent literature has also shown NETs to mediate various other thrombo-occlusive diseases. The ability of platelets to stimulate NET formation will be discussed in Section 2, while the activation of platelets by NETs is the focus of Section 3. An extensive analysis of the literature linking NET formation to vascular disease is presented in Section 4 of this review, and Section 5 describes our current knowledge of pharmacological approaches to control NETs.

## 2. Platelets Stimulate the Formation of NETs

**Figure 2:**
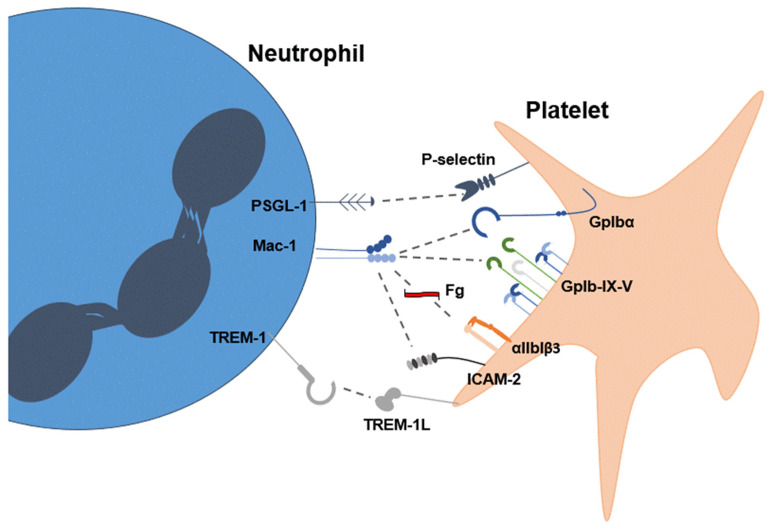
Visual representation of protein–protein interactions allowing platelet–neutrophil aggregation and NET formation. These include P-selectin/P-selectin glycoprotein ligand-1 (PSGL-1), integrin αMβ2 (Mac-1)/glycoprotein 1b alpha (gp1bα), Mac-1/fibrinogen (Fg)/integrin αIIbβ3, Mac-1/intercellular adhesion molecule-2 (ICAM-2), triggering receptor expressed on myeloid cells 1 (TREM-1)/triggering receptor expressed on myeloid cells 1 ligand (TREM-1L).

Platelets have been shown to induce both a slow (suicidal) and a fast (vital) NETosis response in vitro, both of which have been observed in the presence of all classic platelet agonists (thrombin, ADP, collagen, TxA2) [42,43,44]. However, NET formation does not occur in the absence of platelet activation, leading to the consensus that platelets must be activated to induce NETs [15,45,46]. Several receptors on platelets and neutrophils are involved in the formation of platelet–neutrophil aggregates and in the generation of NETs (Figure 2). Direct platelet–leukocyte interactions have been shown to depend on GPIbα expressed on the platelet membrane, which binds to the integrin αMβ2 (or Mac1) on neutrophils [47]. αMβ2 has also been shown to drive platelet–neutrophil interactions via fibrinogen-mediated interactions with the platelet integrin αIIbβ3 [48,49] and via direct interactions with the intercellular adhesion molecule-2 (ICAM-2) [50]. P-selectin (on platelets) has also been shown to play a central role in the formation of platelet–neutrophil aggregates leading to NET formation, with P-selectin glycoprotein ligand-1 (PSGL-1) as a binding partner on neutrophils [15]. Activated platelets expressing P-selectin on their surface bind to PSGL-1 present on the surface of neutrophils, promoting recruitment and creating a proinflammatory environment [51]. PGSL-1 is also the primary P-selectin receptor that facilitates neutrophil aggregation and rolling on endothelial surfaces through activation of the ERK pathway [52,53]. The ERK pathway has been shown in multiple studies to be required to activate NOX2, which is required for NET formation [54]. P-selectin-induced neutrophil activation also stimulates the secretion of cathepsin G (CTSG) and neutrophil elastase (NE), both of which cleave PSGL-1, acting as a negative regulation loop for further activation [55].

Amongst platelet receptors, the major platelet receptor for collagen, GPVI, has been shown to aid local and systemic immune response by promoting platelet–neutrophil aggregate formation and recruitment of neutrophils [56]. The activation of GPVI initiates downstream signalling through the immunoreceptor tyrosine-based activation motif (ITAM), culminating in the activation of phospholipase C and the exposure of P-selectin on the platelet surface [57]. Other platelet stimuli promoting the ability of platelets to induce NETs include lipopolysaccharide (LPS) and thrombin receptor activator peptide 6 (TRAP-6), both of which induce NET in a high mobility group protein B1 (HMGB1)-dependent manner [45]. Platelets also express high mobility group protein B1 (HMGB1), which binds to the receptor for advanced glycation end products (RAGE) on neutrophils alongside TLR4 [58,59]. This induces NET formation but blocks autophagy by inhibiting the depletion of mitochondrial potential, which, in turn, expresses additional HMGB1 from the neutrophil on the extracellular DNA web; this process has been extensively linked with arterial and venous thrombus formation in myocardial infarction patients [45].

The secretion of cytokines and other biochemical signals from the granules of activated platelets can mediate platelet–neutrophil interactions and promote neutrophil binding and activation. To date, although further studies in this area are required, known platelet-derived paracrine signals promoting or facilitating the formation of NETs include thromboxane A2 (TxA2), platelet factor 4 (PF4), regulated on activation, normal T-cell expressed and secreted (RANTES or CCL5), and the chemokine CXCL7 [60,61,62]. Pharmacological studies with thromboxane receptor inhibitors suggest a direct role of platelet-derived TxA2 in venous thrombosis and NET formation [63]. PF4 has been shown to induce neutrophil autophagy and NET formation in patients with deep vein thrombosis and ligation models of venous thrombosis [64]. Similarly to PF4, RANTES (or CCL5) is contained in platelet granules but has little or no function in haemostasis, but has been suggested to play a role in the ability of platelets to induce NETs [65]. CXCL7, released by platelets, is a proinflammatory cytokine that is proteolytically transformed into neutrophil-activating peptide 2 (NAP2) by neutrophils ([66]). The main role of CXCL7 is to act as a chemotactic activator of neutrophils, which is mediated by the receptors CXCR1 and CXCR2 [67,68,69]. It has been demonstrated that the induction of NETs by CXCL7 is dependent on the activation of neutrophil NADPH oxidase 2 [70].

## 3. Platelets Are Activated by NETs

Platelets recognise and are activated by components of NETs. Toll-like receptor 4 (TLR4) is expressed in platelets and can be activated by NET-associated histones [71]. Recent research has shown that TLR4 is essential for platelet activation in an ERK5-GPIIb/IIIa integrin-dependent manner [72]. Interestingly, published data show that histone H4 is the most potent platelet activator via TLR2 and TLR4, with H1, H2A, H2B, and H3 having more modest aggregation profiles [73]. Additionally, histones H3 and H4 can trigger activation of the nod-like receptor protein (NLRP-3) and caspase-1 cleavage in platelets, which may lead to thrombus formation [74]. Recent studies have shown that double-stranded DNA present in NETs is recognised by multiple receptors on platelets, including Toll-like receptor 9 (TLR9) [71], AIM2-like receptors (ALRs), and cyclic GMP-AMP synthase (cGAS) [75], which can lead to platelet activation [76].

NETs can also recruit fibrinogen, which can activate platelets through different mechanisms. In a study by Fuchs et al. [77], the histones on the NETs can recruit fibrinogen to induce platelet activation and aggregation through αIIbβ3. Anchored activated platelets also express phosphatidylserine (PS) on their plasma membrane, which has been demonstrated to facilitate the accretion of coagulation factors on the negatively charged phospholipids [78]. NETs have been shown to promote thrombin generation independently of activated platelets. They activate the coagulation factors XI and XII and can impair the thrombomodulin-dependent protein C activation [73,79]. Studies have also demonstrated that the presence of cell-free DNA (cfDNA) triggers thrombin generation via activation of the contact pathway, with later studies questioning how strong this activation is [79,80,81]. NETs also induce tissue factor pathway inhibitor (TFPI) degradation, which increases coagulation [82,83]. Interestingly, multiple studies have shown that the disassembly of the DNA web using DNase I can significantly reduce the procoagulant effects of NETs [84].

Further interest has been triggered by the ability of NET-associated damage-associated molecular patterns (DAMPs) to induce platelet activation and thrombosis. Proinflammatory proteins S100A8/A9, myeloperoxidase (MPO), elastase, cathepsin G, and the antimicrobial peptide LL-37 fall into this category [78]. The heterodimers of S100A8 and S100A9 are established as markers of NETs [85] and have been shown to regulate the balance between haemostasis and thrombosis in different cardiovascular conditions [86,87]. The direct prothrombotic effect of these proteins on platelets is mediated by glycoprotein 1b alpha (gp1bα) [88]. MPO is a well-recognised component of NETs, and its prothrombotic activity has been reported. MPO interacts with and activates platelets, increasing platelet reactivity and predisposing the cardiovascular system to thrombotic complications [89]. Elastase and cathepsin G have been amply described for their ability to interact and amplify platelet responses [90]. Recent data suggest that the collagen receptor GPVI is the main target for elastase on platelets [91]. Cathelicidins such as LL37 have also been shown to induce platelet activation through the GPVI activation pathway, forming thrombi [92]. Although LL37 is primarily known for its function as an antimicrobial peptide against bacteria, fungi, and viral particles, it also modulates the adaptive immune response, primarily through the formyl peptide receptor 2 (FPR2/ALX) [93]. Interestingly, platelets have been observed to also store LL37 in their granules, a substance which is released following stimulation with TRAP6, CRP-XL, or collagen [94].

A less investigated but extremely interesting mechanism of platelet stimulation by NET involves microparticles (MPs). MPs are released by neutrophils in response to PMA-induced cell activation and NETosis in vitro [95]. Both platelet- and neutrophil-derived MPs have been shown to adhere to histones and accumulate in NETs [96]. MPs trapped in NETs promote thrombin generation, leading to platelet activation and fibrin deposition [97]. This molecular mechanism seems particularly important in prothrombotic complications of infection and sepsis [46,98].

## 4. The Platelet–NET Involvement in Disease

Recent developments in our understanding of immune regulation and pathogen clearance suggest that NETs play a central role in the immune defence. Following infections, NETs remain for multiple days [99]. Although it was initially believed that NETs were degraded by secreted plasma nuclease DNase 1, multiple poorly understood phenomena are also involved [100]. Excessive, unwanted, or persistent NET formation or accumulation drives different pathologies. The activation of neutrophils and platelets and NET formation have been correlated to an increased risk factor for a variety of vascular conditions. A series of conditions in which the interplay of NETs and platelet activation are considered an important etiological component are listed below.

A cardiovascular condition associated with, and potentially facilitated by, NET formation is acute myocardial infarction (AMI). A study by Hally et al. [101] demonstrated the importance of combining the use of NETosis and platelet activation biomarkers for risk prediction in patients with AMI. Platelet-driven thrombosis formation within the coronary artery is the causal factor of ST-elevation myocardial infarction (STEMI), which can lead to acute coronary syndrome (ACS) [102,103]. The standard protocol for STEMI treatment involves antiplatelet therapy; however, the dosage and duration of treatment vary greatly and may result in serious side effects [104]. Following STEMI, NETosis leads to excess thrombin generation, further enhancing platelet activation and inducing further NET formation [105]. The additional thrombin generation also initiates apoptosis in cardiomyocytes and promotes cardiac fibroblast proliferation through interaction with PAR-1 [106]. NET formation to the STEMI pathology contributes to an impaired outcome of this condition, and novel therapeutic approaches are required to control this aspect of the disease [107,108,109].

Reduced blood flow to parts of the brain is the causal factor for ischemic stroke, which prompts a strong inflammatory response associated with intense neurodegeneration and poor disease outcome. Interestingly, the outcome of stroke conditions is closely linked to plasma NET biomarkers. Studies have shown that the upregulation of the NET marker HMGB1 is significantly correlated with the severity of the disease in stroke patients, suggesting that NETs may be a valid target for treatment [110]. In particular, NETs have been shown to promote thrombo-inflammation through the upregulation of citrullinated histone H3 (H3CIT+) and neutrophil granule serine proteases (NSPs) [111,112,113]. The increased PAD4 circulation in stroke patients has also been demonstrated to enhance thrombosis through vWF–platelet interactions and reduce ADAMST13 activity, which is a further suggestion of a role for NETs in this disease [114].

Type 1 diabetes (T1D) is an autoimmune disease in which the beta cells in the pancreas are targeted by the immune system, resulting in hyperglycaemia [115]. Interestingly, circulating NETs are increased in recent-onset T1D [116,117,118]. Studies have shown that neutrophil activation and NET protein marker levels negatively correlate with glycaemic control, which suggests a pathogenic role for neutrophils [119,120]. Similarly, type 2 diabetes mellitus (T2DM) patients exhibit increased levels of NET formation [10,121]. Elevated serum NET levels are a potential serological prognostic marker for T2DM patients at risk of foot-ulcer-related amputation [122]. Taken together, the clinical literature suggests that an increase in thrombo-occlusive events and hypofibrinolytic conditions is the likely cause of increased vascular risk in diabetes patients [123].

Convincing evidence has shown that NETs play a crucial role in sepsis and the resulting organ damage [124]. A significant component of the vascular damage caused by sepsis is mediated by a NET-dependent hypercoagulation state resulting in thrombosis, particularly in the venous circulation [125]. NETs contribute to the thrombotic complications associated with sepsis via three different mechanisms: (1) prothrombotic components of NETs, such as DNA and histone proteins, recruit and activate platelets at the sites of vascular injury [40]; (2) histone binding [126] and neutrophil-derived interleukin-1a (IL-1a) and cathepsin G induce vascular endothelial proinflammatory changes, including the upregulation of vascular cell adhesion molecule 1 (VCAM-1) and intercellular adhesion molecule 1 (ICAM-1) [127]; (3) complement proteins C3 and C5 trapped within NETs activate neutrophils and stimulate the coagulation cascade [128,129], while tissue factor-rich NETs have been shown to drive immune-thrombosis during sepsis [130]. A less investigated mechanism linking thrombosis and NETs involves platelet-derived extracellular vesicles, which have been shown to promote the formation of NETs and organ damage in sepsis [20].

The vascular damage induced by NETosis can be mediated by the effect of NETs on endothelial cell viability and function [131], which ultimately leads to platelet activation and thrombotic disease complications. NETs have been shown to interfere with intercellular junctions and endothelial barrier function [132] and with the anticoagulatory activity of endothelial cells [127,133]. Although some evidence points towards their positive role during the immune response [134], NETs have been shown to contribute to the vascular damage associated with sepsis in both animal models [135] and patients [125]. The vascular complications associated with SARS-CoV-2 infection have been linked to the damaging effect of NETs on endothelial cell function and blood vessel wall integrity [136]. SARS-CoV-2 patients display raised levels of circulating NETs which appear to originate from the lungs [137,138], and the control of NET levels has been proposed to protect vascular health and improve prognosis in these patients [139,140,141].

The role of both platelets and NETs in SARS-CoV-2 infections has been highly investigated due to the extensive reports of neutrophil recruitment to the site of infection resulting in a strong NET response [142]. Neutrophil extravasation has been extensively observed in pulmonary, liver, and myocardial capillaries from SARS-CoV-2 patients [143,144]. SARS-CoV-2 patients have shown an increased fraction of immature neutrophils termed low-density granulocytes (LDGs), which are more likely to undergo spontaneous NET production [145,146,147]. In addition, mature neutrophils with increased CD10 and CD16 expression are also enhanced in SARS-CoV-2 patients, which is likely to cause NETosis [148]. Importantly, autoimmune antibodies against phospholipids, phospholipid-binding proteins, and PF4 are increased by SARS-CoV-2 infection, which can induce platelet activation and NET formation [149,150,151]. This process can generate microthrombi and has been observed to occur in some patients in response to vaccination, causing vaccine-induced thrombotic thrombocytopenia (VITT) [152].

## 5. Pharmacological Approaches Targeting NETs

With many studies demonstrating the contribution of NETs [153] and their stimulation by platelets to vascular conditions [154], there is growing interest in developing effective pharmacological strategies to reduce or control NET formation. Investigation into microbial resistance to NETs is a growing research field, which aims to develop new strategies for anti-inflammatory agents or new drugs that inhibit NETs using our understanding of how some pathogens can evade NETs [155]. Despite multiple reports of different plant bioactive compounds able to combat NETs in the treatment of inflammation, due to the complex nature of these compounds, the identification of their mechanism of action and the development of novel pharmacological interventions based on them remains challenging [156,157,158].

The use of DNase I has already been approved for the treatment of cystic fibrosis and SLE, both of which have since been shown to have extensive NET involvement [159,160]. A recent study by Englert, et al. [161] demonstrates that combining DNase I and DNaseIL3 significantly increase NET degradation. This has been observed both in in vitro human NETs and in knockout mice. Additionally, a recent study by Fisher, et al. [162] has shown encouraging results in a small sample of patients with acute COVID-19 treated with aerosolised DNase I. Similar data were obtained by Veras, et al. [163]. For this reason, DNase-based pharmacological treatments are currently under intense investigation [164].

The blockade of the gasdermin D (GSDMD) pathway would reduce the amount of NE translocating to the nucleus to begin NETosis, alongside the reduction in the formation of pores in the plasma membrane. The FDA has approved the use of disulfiram, which inhibits GSDMD and pyroptosis, which, by extension, has been shown to inhibit NET formation and protect rodents from COVID-19 [165,166]. A study by Yang, et al. [167] has suggested the use of disulfiram as a treatment for diabetic foot ulcers that acts by blocking NET formation. Interestingly, a small molecule inhibitor of GSDMD called LDC7559 has been shown to inhibit PMA-induced NETosis efficiently and shows some promise for the development of a NET-targeting treatment [168].

Plasma MPO levels are a marker of NET formation and a marker for poor prognosis [150]. Importantly, the inhibition of MPO by AZM198 has been demonstrated to effectively reduce NET formation both in vitro and in vivo [169]. Moreover, the peptide inhibitor of complement C1 PA-dPEG24 has been shown to dose-dependently inhibit NET formation [170]; its mechanism of action depends on the inhibition of the peroxidase activity of MPO [171]. A recent study has shown that PA-dPEG24 is safe and well tolerated for the reduction of neutrophil-mediated inflammation in humans induced by inhaled LPS challenge, proposing it as a potential new therapeutic agent [172].

The generation of ROS has been shown to play a key role in the formation of NETs. This has been shown, for example, in patients of SARS-CoV-2, where free radicals seem responsible for the uncontrolled NET formation and T-cell activation that results in the extensive inflammation associated with the disease [173]. Although multiple herbal medicines have been shown to inhibit ROS (e.g., vitamin C, luteolin, and N-acetyl-cysteine (NAC)), their testing as NET-targeting agents has led to inconclusive results [153,174]. Nonetheless, the targeting of NETs via inhibition of NADPH oxidases and Rag-MEK-ERK signalling pathways has been proposed [13,175]. Interestingly, the standard diabetes treatment metformin has been shown to diminish NET formation via a reduction of the activation of NADPH oxidases [176].

The role of platelets in stimulating NETs also offers some pharmacological opportunities. Stachydrine extracted from motherwort has been shown to suppress platelet activation and decrease platelet–neutrophil interactions [177]. The use of antiplatelet therapies such as ticagrelor has also been shown to reduce NET markers in patients with pneumonia, highlighting that, although large amounts of research focus on NETs specifically, their interaction with platelets and other cell types should be more thoroughly investigated [178,179]. For example, a study by Wallis et al. highlights that the Staphylococcus aureus extracellular fibrinogen-binding protein (Efb) Efb_68–87_ binds directly to platelets and inhibits their P-selectin interaction with leukocytes, leading to decreased NET formation in vitro (Wallis, Wolska, Englert, Posner, Upadhyay, Renné, Eggleston, Bagby and Pula [44]). This and similar agents deserve further investigation.

Since the drug discovery for novel NET inhibitors is still in its infancy, several opportunities are available. A collection of pharmacological agents currently used for treating a variety of illnesses has shown the ability to control inflammation via the inhibition of NET formation. One such agent is benserazide, an inhibitor of the dopamine pathway that impairs NET formation through a mechanism that remains to be elucidated but which has shown some potential for drug repurposing [180]. Moreover, a study by Du, et al. [181] demonstrates that S100A9 induces ROS-dependent formation of NETs via TLR4 and RAGE signalling, which can be inhibited by using ABR-238901. The heterogeneity of signalling pathways leading to NET formation offers multiple targeting opportunities. This concept is reinforced by Keir, et al. [182], who found that targeting neutrophilic inflammation with existing antibiotics such as azithromycin reduces NET markers in the sputum of bronchiectasis patients.

In addition to the promising pre-clinical or early clinical studies listed above, some therapeutic agents in clinical use have been shown to achieve their effect via inhibition of NETs in patients. One example is macrolide antibiotics, which have been shown to achieve their therapeutic effect, at least partially, via the inhibition of NETs in pneumonia patients [183]. Similarly, the antimalarial drugs chloroquine and hydroxychloroquine have been shown to significantly protect against venous thrombosis in cancer patients [184]. Several non-antibiotic drugs currently approved for clinical use have also been shown to effectively reduce NET formation in patients. Ticagrelor [178], aspirin [185], and dipyridamole [186,187] have been shown to inhibit NET formation by reducing platelet activation and platelet-dependent NET stimulation. Similarly, crizanlizumab has been shown to significantly reduce NET formation in patients via the inhibition of P-selectin and the abolishment of platelet– and endothelial-cell–neutrophil interactions [188]. Several anti-inflammatory drugs targeting interleukins and other inflammatory cytokines have been shown to achieve their therapeutic effect also via a significant reduction of NET formation. These pharmacological agents include canakinumab [189], infliximab [190], and tocilizumab [191]. Finally, some approved pharmacological agents that target components of NETs or the molecular mechanisms leading to their formation are beneficial for patients via the inhibition of NET-induced cytotoxicity. These include a recombinant human soluble thrombomodulin that neutralises histone cytotoxicity called ART123 [192], the gasdermin D blocker disulfiram [165], the DNA-degrading enzyme dornase alpha [193], and the elastase inhibitor sivelestat [194].

## 6. Conclusions and Future Perspectives

The ability of neutrophils to release DNA-rich extracellular traps is very important for the innate immune response, and the investigation of this phenomenon has become central to modern vascular biology. NETs entrap platelets and other blood cells to promote thrombosis and, as a result, in addition to protecting from infection, they have been indicated as a cause of vascular inflammation and thrombosis in different health conditions. Despite their importance, the molecular mechanisms responsible for platelet-dependent NET formation are only partially understood, limiting the ability to target this pathological response to protect vascular health. In this review, we describe our current understanding of NETs and their reciprocal regulation with platelets which has the potential to become an important drug discovery target in vascular medicine. The growing interest in this phenomenon and the methodological advances of recent years suggest that novel drug interventions targeting NETs will appear in the near future and will have the ability to have an important impact on cardiovascular medicine. 

## Data Availability

No new data were created or analyzed in this study.
